# Relationship Between Quadriceps Muscle Strength Asymmetry and Lower Limb Biomechanical Asymmetry During Running in Patients Who Underwent Anterior Cruciate Ligament Reconstruction

**DOI:** 10.3390/bioengineering13040400

**Published:** 2026-03-30

**Authors:** Xialin Ge, Mingxuan Gao, Yiming Tao, Longting Suo, Shuang Ren, Yingfang Ao

**Affiliations:** 1 Tianjin Key Laboratory of Exercise Physiology and Sports Medicine, Institute of Sport, Exercise & Health, Tianjin University of Sport, Tianjin 300381, China; gxl990829@163.com (X.G.); gmx15831545021@163.com (M.G.); 18596128002@163.com (Y.T.); 19025280592@163.com (L.S.); 2 Department of Sports Medicine, Peking University Third Hospital, Institute of Sports Medicine of Peking University, Beijing 100191, China; 3 Beijing Key Laboratory of Research and Translation for Drugs and Medical Devices in Precision Diagnosis and Treatment of Sports Injuries, Beijing 100191, China; 4 Engineering Research Center of Sports Trauma Treatment Technology and Devices, Ministry of Education, Beijing 100191, China

**Keywords:** anterior cruciate ligament reconstruction, running, quadriceps strength asymmetry, biomechanics

## Abstract

(1) Background: Postoperative anterior cruciate ligament reconstruction often involves quadriceps strength asymmetry, leading to abnormal lower limb biomechanics during running. While previous studies have examined the relationship between isokinetic strength and walking or jumping, the association between running, a key criterion for return to sport, and lower limb biomechanics remains unclear, particularly regarding isokinetic strength asymmetry at different angular velocities. (2) Methods: Isokinetic quadriceps strength, running kinematic, and kinetic data were collected from 39 ACLR individuals. Paired *t*-tests compared bilateral differences, and Pearson correlation analysis assessed associations between biomechanical parameters and muscle strength. (3) Results: The injured leg showed significantly weaker Qc at 60°/s, 180°/s, and 300°/s (*p* < 0.05). Compared to the uninjured leg, the injured leg demonstrated a significantly greater hip flexion angle at initial contact (*p* < 0.05); the injured leg exhibited significantly reduced knee flexion angle at the time of peak vertical ground reaction force and peak knee flexion angle (*p* < 0.05); the injured leg exhibited significantly reduced knee flexion moment at PVGRF, peak knee flexion moment, peak knee extension moment (*p* < 0.05). Both the 60°/s Qc and Qe showed moderate negative correlations with knee flexion angles, and 180°/s Qc correlated with knee flexion moment at PVGRF (*p* < 0.05). (4) Conclusions: ACLR patients show quadriceps strength asymmetry and abnormal sagittal knee and hip biomechanics during running. Strength symmetry moderately correlates with knee kinematics and kinetics in a velocity-dependent manner. Rehabilitation should focus on multi-speed and eccentric training with neuromuscular and hip–knee coordination exercises to optimize movement and support safe return to sports.

## 1. Introduction

The anterior cruciate ligament (ACL) is a critical structure for maintaining dynamic knee stability. Injury to the ACL severely disrupts the mechanical equilibrium of the lower limb kinetic chain, leading to impaired athletic performance. Anterior cruciate ligament reconstruction (ACLR) is the primary surgical intervention to restore knee stability [1]. However, despite standardized rehabilitation, persistent neuromuscular deficits and movement asymmetries of the lower limbs are frequently observed after ACLR. These deficits, particularly quadriceps weakness, are associated with abnormal gait patterns, increased risk of secondary injury, and the development of early knee osteoarthritis [2]. Notably, the prevalence of knee osteoarthritis may reach up to 80% within 10–15 years following ACLR in patients with concomitant meniscal injury [3].

Running represents a critical milestone for returning to sports and high-demand daily activities, as it places substantially greater demands on lower-limb impact loading, neuromuscular coordination, and inter-muscular synergy compared to low-impact tasks such as walking. Previous studies have reported that individuals after ACLR often demonstrate persistent biomechanical asymmetries during running, which are considered important risk factors for secondary ACL injury and long-term joint degeneration [4]. Both gait asymmetry and quadriceps strength deficits have been linked to increased injury risk and the progression of post-traumatic osteoarthritis [5,6,7]. For instance, Paterno et al. [8] reported that athletes who returned to sport with persistent hip and knee biomechanical asymmetries were more than three times more likely to sustain a second ACL injury. Similarly, Roewer [9] and Curran et al. [10] found that hip and knee biomechanical asymmetries persisted at 6 months and even 2 years after ACLR despite improvements in quadriceps strength, suggesting that neuromuscular recovery may be incomplete. These findings highlight the importance of understanding the relationship between muscle strength symmetry and lower-limb biomechanics during dynamic activities such as running.

The quadriceps femoris, as the primary muscle group responsible for knee joint movement and stability, plays a critical role during the deceleration phase of running. During typical rearfoot-strike running, eccentric knee extensor activity contributes substantially to shock absorption and lower-limb energy dissipation [11]. However, most previous studies assessing quadriceps function after ACLR have focused on isometric strength or isokinetic strength at a single angular velocity (e.g., 60c) [2,12,13]. Therefore, whether quadriceps strength asymmetry at different angular velocities differentially influences running biomechanics remains unclear, limiting our understanding of velocity-specific strength deficits and their implications for post-ACLR rehabilitation strategies.

Therefore, this study aimed to investigate the following in individuals who had undergone ACLR: (1) asymmetry in hip and knee joint kinematics during running, and (2) the association between quadriceps strength symmetry assessed at multiple angular velocities (60°/s, 180°/s, and 300°/s) and lower limb biomechanical symmetry. We hypothesized that significant asymmetries would exist between the injured and uninjured limbs in hip and knee biomechanics during running, and that quadriceps strength asymmetry would be associated with lower-limb biomechanical asymmetry.

## 2. Materials and Methods

### 2.1. Participants

All patients met the following inclusion criteria: (1) First-time autologous hamstring tendon graft for anterior cruciate ligament reconstruction; (2) Absence of moderate-to-severe articular cartilage defects in both patellofemoral and tibiofemoral joints. Exclusion criteria: (1) Concurrent other ligament injuries in the knee; (2) History of surgery or musculoskeletal injury in the contralateral knee. All patients were diagnosed with ACL rupture via arthroscopy and underwent unilateral autologous hamstring tendon single-bundle ACLR. The femoral bone track was stabilized using Endobutton (Smith & Nephew, Memphis, TN, USA), while the tibial bone tunnel employed compression fixation with interference screws (Smith & Nephew, USA). Additionally, all ACLR subjects followed a standardized home rehabilitation protocol. Postoperative isometric contraction training for the quadriceps and hamstrings, along with straight leg raises, commenced on day 1. Patients wore braces and used crutches for ambulation, with partial weight-bearing initiated within 2 weeks postoperatively and full weight-bearing thereafter. Range of motion (ROM) for flexion and extension was increased to 90° by days 3–7 postoperatively and to 120° by week 4. Closed-chain exercises began at 6 weeks postoperatively. Prior to analysis, a sample size estimation was performed using G*Power software (version 3.1). Based on an expected effect size of Cohen’s d = 0.80 for paired comparisons, a significance level of α = 0.05 (two-tailed), and a desired statistical power of 0.80 [14], the calculation indicated that 26 participants were required. To ensure adequate power and account for potential attrition, 39 participants with ACLR were ultimately recruited. The use of human participants in this study was approved by the Institutional Review Board of Peking University Third Hospital (No. M20250039). All participants read and signed a written informed consent before data collection.

### 2.2. Data Collection

Using an established plug-in gait model, reflective markers were placed on the following anatomical landmarks: bilateral anterior superior iliac spines (ASIS), posterior superior iliac spines (PSIS), mid-thigh (at 50% of the thigh length), lateral distal thigh, medial and lateral femoral epicondyles, proximal and distal thirds of the tibia, lateral distal tibia, lateral and medial malleoli, calcaneus, and the first, second, and fifth metatarsophalangeal joints. Subjects wore athletic shorts and performed the test at a self-perceived comfortable jogging pace [15]. If a subject’s running speed showed significant fluctuations compared to previous valid trials, exhibited suspected head-down glancing at the force plate, or failed to meet the test requirement of stepping on both force plates twice with each lower limb, the trial was repeated [15]. To ensure feet were accurately centered on the force platform and minimize variability, subjects completed at least three successful practice trials before formal data collection. Each subject underwent five trials, with at least three valid test data points retained for analysis. A 12-camera motion capture system (Vicon Nexus software, version 1.8.5; Vicon Motion Systems Ltd., Yarnton, UK) recorded the three-dimensional trajectories of the reflective markers at 100 Hz. Ground-reaction force (GRF) data were synchronously collected using two embedded force plates (AMTI, Watertown, MA, USA) sampled at 1000 Hz.

After the running test, an isokinetic force test was performed using an isokinetic dynamometer (CON-TREX MJ, version MK2m, Physiomed Elektromedizin AG, Schnaittach, Germany) with a sampling frequency of 256 Hz. The subject sits in a fixed position, and the torso and the test thigh are firmly bound to the test equipment. The range of motion of the knee joint is set at 90° to 20° of flexion. Gravity effect torque was recorded for each patient over the entire range of motion and used for correction of torque measurements in all isokinetic tests [16]. Before the formal test, the patient tried to do 1 or 2 simulated actions, familiarize himself with the test process and the timing of exerting force, and rest for 90 s between the two tests. The tests were performed in the order of uninjured limbs first and injured legs later. Each limb was sequentially tested for centripetal knee extension muscle strength at 60°/s, 180°/s, 300°/s, and eccentric knee extension muscle strength at 60°/s [17,18,19]. During the test, the subjects were instructed to complete 5 knee extension and flexion exercises with the maximum force, and exerted force as quickly as possible during the knee extension stage. The isokinetic peak torque mean (in Nm) was calculated after the test and normalized to body weight (in Nm/kg); a 3 min rest interval was set between formal tests with different angular velocities and contraction patterns to eliminate the influence of muscle fatigue on the test results.

### 2.3. Data Reduction

The raw marker trajectory data and ground reaction force (GRF) data were low-pass filtered using a fourth-order zero-lag Butterworth filter with cutoff frequencies of 12 Hz and 75 Hz [20], respectively. This filtering configuration effectively removes high-frequency noise while preserving the original dynamic characteristics of the kinematic and GRF signals, thereby preventing waveform distortion from compromising the accuracy of joint moment calculations via inverse dynamics. The motion capture system and force plate in this study were synchronized via hardware connection, initiated by a single trigger signal for data acquisition. Subsequent frame synchronization calibration was performed using Visual 3D software (C-Motion, Germantown, MD, USA; Version 6.01.0), ensuring complete temporal alignment between kinematic and ground reaction force data with no time offset. Segment coordinate systems were defined for the thigh and shank. All joint moments reported in this study are internal moments. Joint angles were calculated in Cartesian coordinates with the following sequence: (1) Flexion–Extension (x-axis), (2) Abduction–Adduction (y-axis), (3) Internal–External Rotation (z-axis). Joint angles were calculated in Cartesian coordinates with the following rotation sequence: (1) flexion–extension (x-axis), (2) abduction–adduction (y-axis), (3) internal–external rotation (z-axis). This sequence describes the orientation of the distal segment relative to the proximal segment. Joint moments were calculated using an inverse dynamic approach and transferred into the distal segment reference frame. GRFs were normalized to the body weight (BW). Joint moments and muscle strength were normalized to the production of BW (measured in newtons) and standing height (BH; measured in meters) and calculated as BW·BH [14]. All variables were analyzed during the stance phase. Initial contact (IC) was defined as the first frame at which the vertical GRF exceeded 10 N; toe-off (TO) was defined as the frame after IC at which the vertical GRF fell and remained below 10 N.

The quadriceps strength index (QI) is calculated as follows: QI=(Injured leg/Uninjured leg)×100% [14]. Among them, a QI value of 100% indicates that the strength of both limbs is completely symmetrical, and a QI value below 100% indicates that the affected limbs have strength deficiency [21]. Interlimb differences in lower limb joint biomechanics were calculated to investigate left-right symmetry during running. The inter-leg difference (ILD) between the injured and uninjured legs was computed based on joint angles and moments using the following method: ILD=Uninjured leg−Injured leg [12]. A positive value of ILD indicates that the value of the injured leg is smaller than that of the uninjured leg, and a negative value of ILD is the opposite.

The multiple phases of the running stance were analyzed [22]. Initial contact is characterized by eccentric contraction of the quadriceps, marking the instant when the foot first strikes the ground and the body begins to absorb impact. The peak vertical ground reaction force typically occurs during mid-stance, corresponding to both the maximum vertical loading and the greatest knee flexion angle. Finally, the heel-off phase extends from heel rise until toe-off, during which the body’s center of mass accelerates forward and upward to generate propulsive force.

### 2.4. Data Analysis

All continuous variables were expressed as mean ± SD. The normality of the data was assessed using the Shapiro–Wilk test. The paired *t*-test was used to compare the differences in biomechanical variables between the injured side and the healthy side. Pearson’s correlation coefficient was used to analyze the correlation between quadriceps strength symmetry index (QI) and biomechanical symmetry index during running at different angular velocities (60°/s, 180°/s, 300°/s). The explanatory criteria for correlation coefficient (r) are: 0–0.1 is trivial, 0.1–0.3 is weak, 0.3–0.5 is moderate, and >0.5 is strong. Given the exploratory nature of the correlation analysis and the involvement of multiple biomechanical variables, no *p*-value adjustment was performed for multiple comparisons. In hypothesis-generative studies, rigorous family error rate corrections (such as Bonferroni corrections) may increase the risk of Class II errors, thus masking potentially biologically meaningful associations, and therefore relevant results need to be interpreted with caution. Effect sizes for paired comparisons were calculated using Cohen’s d and interpreted by the following criteria: small (<0.20), small (0.20–0.49), moderate (0.50–0.79), and large (≥0.80) [23]. All statistical analyses were performed using SPSS software (version 27.0; IBM Corporation, Armonk, NY, USA) and the level of statistical significance was set at *p* < 0.05 (two-tailed test).

## 3. Results

A total of 39 patients ultimately met the inclusion criteria for this study (Table 1).

### 3.1. Quadriceps Muscle Strength

The Qc of the injured leg was significantly weaker at 60°/s, 180°/s, and 300°/s compared with the uninjured leg (*p* = 0.002, Cohen’s d = 0.54; *p* < 0.001, Cohen’s d = 0.66; *p* < 0.001, Cohen’s d = 0.76) (Table 2).

### 3.2. Kinematics and Kinetics

Compared to the uninjured leg, the injured leg demonstrated a significantly greater hip flexion angle at initial contact (*p* = 0.011, Cohen’s d = 0.43). Compared to the uninjured leg, the injured leg exhibited significantly reduced knee flexion angle at the time of peak vertical ground reaction force (*p* = 0.006, Cohen’s d = 0.47) and peak knee flexion angle (*p* < 0.001, Cohen’s d = 0.59) (Table 3).

Compared with the uninjured leg, the injured leg exhibited significantly reduced knee flexion moment at PVGRF (*p* < 0.001, Cohen’s d = 0.59), peak knee flexion moment (*p* < 0.001, Cohen’s d = 1.11), peak knee extension moment (*p* = 0.011, Cohen’s d = 0.43), and PVGRF (*p* < 0.001, Cohen’s d = 0.64) (Table 4).

### 3.3. Correlation Between Quadriceps Strength Symmetry and Lower Limb Biomechanical Asymmetry

The 60°/s Qc showed moderate negative correlations with knee flexion angle at PVGRF (r = −0.33, *p* = 0.041) and peak knee flexion angle (r = −0.39, *p* = 0.013). The 180°/s Qc demonstrated a moderate negative correlation with knee flexion moment at PVGRF (r = −0.38, *p* = 0.017). The 60°/s Qe exhibited moderate negative correlations with knee flexion angle at PVGRF (r = −0.43, *p* = 0.006) and peak knee flexion angle (r = −0.39, *p* = 0.014). All other correlations were weak or negligible and did not reach statistical significance (*p* > 0.05) (Table 5).

## 4. Discussion

This study explored the relationship between quadriceps strength asymmetry and lower extremity biomechanical asymmetry during running in patients after ACLR. Overall, the main findings partially support our hypothesis. Significant inter-limb differences were found in quadriceps strength at multiple angular velocities and in kinematics and dynamics of the hip and knee joints of the injured limb. Furthermore, moderate correlations were observed between quadriceps strength symmetry and knee sagittal biomechanical parameters, and these associations were velocity-dependent. This core finding suggests that the rehabilitation training after ACLR should include multi-speed muscle strength training and eccentric strength training, so as to more fully restore the dynamic function of knee joint during running. Given the cross-sectional design, these findings should be interpreted as hypothesis-generating rather than definitive causal conclusions.

### 4.1. Differences in Quadriceps Strength Between Limbs After ACLR

Quadriceps weakness is a common neuromuscular deficit following ACLR. In this study, the Qc of the injured leg at 60°/s, 180°/s, and 300°/s was significantly lower than that of the uninjured leg. Previous studies have reported that reduced quadriceps strength is associated with altered movement patterns after ACLR [24], muscle atrophy [25], and changes in sensory input [26], and this deficit may increase the risk of secondary injury [27]. A cross-sectional study demonstrated that quadriceps isokinetic strength at 9 and 12 months post-ACLR remained significantly lower than at 24 months, reflecting the long-term nature of strength recovery [28]. Palmieri-Smith et al. [29] further indicated that persistent deficits in quadriceps neural activation exist after ACLR. Even when knee structures have healed, the neural drive to the muscle may not fully recover, and this neuromuscular control deficit is a key mechanism underlying long-term strength loss. In addition, factors such as pain avoidance after surgery and insufficient eccentric training during rehabilitation may exacerbate the strength deficit of the injured quadriceps. Therefore, post-ACLR rehabilitation should prioritize quadriceps strength recovery, with particular emphasis on balanced improvement across multiple angular velocities. Targeted training to enhance neuromuscular activation efficiency and reduce inter-limb strength asymmetry can help patients safely return to sports and reduce the risk of secondary injury.

### 4.2. Bilateral Lower Limb Biomechanical Differences During Running After ACLR

Abnormal lower limb biomechanics and inter-limb asymmetry during running are risk factors associated with secondary ACL injury and the development of knee osteoarthritis. Shahbazi et al. [30] reported that during overground running, individuals in the ACLR group exhibited greater hip flexion angles compared to the uninjured leg and control limbs. This finding is consistent with our results and may reflect a compensatory mechanism of the gluteus maximus in response to quadriceps weakness [31]. Although such compensation helps maintain forward propulsion during running, the increased hip joint load over time may be associated with a higher risk of overuse injuries and hip osteoarthritis [32,33]. Perraton [34] and Knurr et al. [35] found that during the stance phase of running after ACLR, the knee flexion angle of the operated limb was significantly smaller than that of the uninjured limb. Reduced knee flexion is a common compensatory pattern following ACLR, often arising from quadriceps weakness, impaired neuromuscular control, and compromised knee function [36,37]. This pattern may reflect a quadriceps avoidance strategy, in which individuals reduce knee loading during dynamic tasks to compensate for insufficient quadriceps function and protect the reconstructed knee joint. These abnormal biomechanics may impair the shock-absorbing function of the knee, increase intra-articular stress, and alter normal cartilage load distribution, which is thought to be associated with the pathological processes underlying postoperative secondary joint degeneration [38,39].

This study also provides evidence that dynamic changes after ACLR primarily occur in the sagittal plane. Perraton et al. [34] reported that reduced knee flexion moments after ACLR were closely associated with poorer knee functional outcomes. Pairot [40] and Shi et al. [14] observed that the operated limb demonstrated decreased knee extension moments and peak vertical ground reaction forces compared to the uninjured limb, which may result from a compensatory unloading strategy during dynamic tasks. Psychological factors, such as reduced confidence in the reconstructed leg, may also contribute. Other studies have indicated that increased hip and trunk flexion accompanies reduced knee flexion moments, which may represent part of a quadriceps avoidance mechanism [41]. Overall, abnormal biomechanics are common after ACLR and involve coordinated changes across the hip and knee joints. Therefore, rehabilitation assessment and training should not only focus on local knee strength but also integrate hip–knee coordination and movement strategy training to optimize overall running biomechanics. Given the cross-sectional design of this study, the causal relationship between these biomechanical alterations and strength deficits cannot be determined, and future longitudinal studies are needed to further explore how specific movement pattern changes affect long-term joint health.

### 4.3. Association Between Sagittal Plane Knee Kinematic Asymmetry and Quadriceps Strength Asymmetry

Correlation analysis in this study showed that both 60°/s Qc and Qe were moderately negatively correlated with knee flexion angles at PVGRF and peak knee flexion, indicating that symmetry in slow-speed concentric and eccentric quadriceps strength is associated with bilateral balance of sagittal plane knee kinematics during running after ACLR. During the stance phase of running, relatively slow eccentric and isometric contractions occur, requiring sufficient quadriceps force to control knee flexion and absorb impact [11]. Notably, insufficient eccentric function may directly impair the shock-absorbing capacity of the knee [42]. These findings are consistent with previous studies demonstrating that quadriceps strength symmetry is closely associated with symmetrical knee kinematics during dynamic tasks [12]. Palmieri-Smith et al. reported that patients with lower quadriceps symmetry exhibited lower symmetry in knee flexion angles during landing compared to patients with higher quadriceps symmetry [43]. In contrast, 180°/s and 300°/s Qc were not significantly correlated with knee kinematic parameters, which may reflect the mismatch between high-speed muscle strength testing and the actual demands of running. Although running involves rapid movements, the stance phase primarily requires controlled force output rather than maximal velocity contractions. It is noteworthy that all significant correlations observed in this study were weak to moderate, suggesting that quadriceps strength asymmetry can only partially explain kinematic variation, and other factors such as neuromuscular coordination and movement strategies are also important. These findings highlight the importance of slow-speed and eccentric quadriceps training during ACLR rehabilitation. Such training may help restore knee joint loading symmetry and improve dynamic knee stability during running. Furthermore, targeted neuromuscular training to improve quadriceps strength symmetry [44] may optimize sagittal plane knee kinematics and contribute to long-term knee joint health.

### 4.4. Association Between Sagittal Plane Knee Kinetic Asymmetry and Quadriceps Strength Asymmetry

In this study, 180°/s Qc was moderately negatively correlated with knee flexion moment at PVGRF, suggesting that quadriceps strength symmetry may be associated with sagittal plane knee kinetics during running. During the landing phase of running, the quadriceps primarily control knee flexion and regulate joint moments through eccentric contraction, helping to absorb ground reaction forces and stabilize the knee [22]. When quadriceps strength is insufficient, patients may reduce knee flexion or alter moment patterns to decrease knee load; although this compensatory strategy may provide short-term protection, it may lead to long-term alterations in joint mechanics. Previous studies have shown that ACLR patients with quadriceps weakness often exhibit decreased knee moments and altered sagittal plane kinetic patterns [12,45], particularly during running or weight-bearing gait tasks. Furthermore, quadriceps strength levels are closely related to knee flexion and extension moments, and insufficient strength can affect load distribution and motor control at the knee [29,46]. Therefore, these findings indicate that quadriceps strength restoration should be emphasized during ACLR rehabilitation to improve sagittal plane knee kinetics and facilitate a safe return to running.

Collectively, these findings highlight the importance of restoring quadriceps strength symmetry across multiple contraction velocities to optimize lower-limb biomechanics and facilitate safe return to running after ACLR.

## 5. Clinical Implications

This study suggests that quadriceps strength asymmetry in patients following ACL R is associated with sagittal plane kinematics and dynamics during running, and this association exhibits speed dependency. Clinical rehabilitation should incorporate multi-angular velocity and eccentric strength training while emphasizing neuromuscular control and hip–knee coordination training [44,47,48]. This approach aims to enhance knee dynamic stability and movement control patterns, facilitate safe return to sports, and reduce the risk of potential secondary injuries.

## 6. Limitations

There are several limitations in this study, which may affect the comprehensiveness of the findings and the extrapolation of the conclusions. First, a causal relationship between quadriceps strength symmetry and running biomechanics could not be established due to the cross-sectional design of the study. Second, the study did not set up a healthy control group, and it was difficult to judge whether the observed biomechanical asymmetry was a specific manifestation after ACLR. Third, biomechanical analysis is limited to the sagittal parameters of hip and knee joints, and lacks coronal, cross-sectional data and electromyographic recordings, so it is impossible to comprehensively analyze the neuromuscular regulatory mechanism behind lower limb movement asymmetry. Fourth, the research sample size is relatively limited, and it is difficult to fully control the interference of potential confounding factors such as gender and running speed on the results. Fifth, since multiple correlations were explored, the possibility of class I errors could not be ruled out. Therefore, these findings should be interpreted with caution and need to be validated in future studies with larger samples. Future studies can adopt a longitudinal design, include healthy controls, collect multiplanar biomechanical and electromyographic data, and expand the sample size for subgroup analysis to further verify and improve the results of this study.

## 7. Conclusions

After ACLR, quadriceps strength asymmetry and biomechanical abnormalities of the knee and hip joint were present in the sagittal plane during running. Quadriceps strength symmetry was moderately correlated with knee kinematics and dynamic parameters and showed velocity dependence. The results suggest that in the rehabilitation after ACLR, attention should be paid to multi-angular velocity and eccentric muscle strength training, combined with neuromuscular control and hip–knee synergistic training, to optimize the knee movement pattern and promote safe return to exercise. Because of the cross-sectional design of this study, these findings are mainly associative suggestions, and their effects on long-term joint health and risk of re-injury need to be further validated by longitudinal studies.

## Figures and Tables

**Table 1:** Participant Characteristics ^a^.

Characteristic	ACLR Group, Mean ± SD
Age, y	32.97 ± 6.52
Sex (male/female)	22/17
Body mass, kg	75.14 ± 12.75
Body height, m	1.73 ± 0.08
BMI, kg/m^2^	25.02 ± 2.74
Time since surgery, mo	9.2 ± 0.7
Pre-injury Tegner score	6.67 ± 1.46
IKDC 2000	63.13 ± 9.49
Lysholm score	80.31 ± 14.08
Injury side (left/right)	18/21
Gait speed (m/s), m	2.51 ± 0.24

^a^ ACLR: anterior cruciate ligament reconstruction; BMI: Body Mass Index; IKDC 2000, International Knee Documentation Committee Subjective Knee Form.

**Table 2:** Peak isokinetic quadriceps strength.

Variables	Uninjured Leg	Injured Leg	QI (%)	*p* Value	Effect Size (Cohen’s d)	95%CI
Qc (60°/s), Nm/kg	1.58 ± 0.49	1.32 ± 0.59	83.44 ± 28.46	0.002 *	0.54	−0.409, −0.101
Qc (180°/s), Nm/kg	1.35 ± 0.39	1.09 ± 0.47	80.78 ± 27.40	<0.001 *	0.66	−0.380, −0.131
Qc (300°/s), Nm/kg	0.93 ± 0.40	1.17 ± 0.36	80.30 ± 25.56	<0.001 *	0.76	−0.335, −0.135
Qe (60°/s), Nm/kg	1.80 ± 0.61	1.65 ± 0.69	92.89 ± 29.15	0.091	0.28	−0.316, 0.025

* *p* < 0.05, reflecting interlimb differences. QI, Quadriceps Strength Index; Qc, concentric quadriceps strength; Qe, eccentric quadriceps strength.

**Table 3:** Selected Kinematic parameters of the hip and knee joints during running (mean ± standard deviation) and bilateral differences (ILD) between the uninjured and injured legs.

Variables	Uninjured Leg	Injured Leg	ILD	*p* Value	Effect Size (Cohen’s d)	95% CI
Knee flexion angle at IC, °	26.32 ± 5.48	25.71 ± 4.39	0.61 ± 4.22	0.370	0.15	−1.980, 0.755
Hip flexion angle at IC, °	46.27 ± 7.71	47.83 ± 8.01	−1.56 ± 3.64	0.011 *	0.43	0.380, 2.738
Knee flexion angle at PVGRF, °	39.65 ± 5.81	36.85 ± 5.05	2.80 ± 6.02	0.006 *	0.47	−4.753, −0.851
Hip flexion angle at PVGRF, °	36.81 ± 9.86	37.26 ± 8.84	−0.45 ± 4.20	0.508	0.11	−0.911, 1.810
Knee flexion angle at TO, °	34.68 ± 7.42	34.22 ± 7.79	0.46 ± 8.75	0.743	0.05	−3.298, 2.372
Peak knee flexion angle, °	42.07 ± 5.57	38.47 ± 4.81	3.60 ± 6.14	<0.001 *	0.59	−5.591, −1.610

* *p* < 0.05, reflecting interlimb differences. ILD, inter-leg difference, a positive value indicates the uninjured leg > injured leg; a negative value indicates the injured leg > uninjured leg; IC, initial contact; PVGRF, peak vertical ground reaction force; TO, toe-off.

**Table 4:** Selected kinetic parameters of the hip and knee joints during running (mean ± standard deviation) and bilateral differences (ILD) between the uninjured and injured legs.

Variables	Uninjured Leg	Injured Leg	ILD	*p* Value	Effect Size (Cohen’s d)	95% CI
Hip extension moment at IC, BW·BH	0.020 ± 0.014	0.021 ± 0.010	−0.001 ± 0.012	0.720	0.06	−0.005, 0.003
Knee flexion moment at PVGRF, BW·BH	0.122 ± 0.033	0.099 ± 0.030	0.023 ± 0.038	<0.001 *	0.59	−0.035, −0.010
Hip flexion moment at PVGRF, BW·BH	0.030 ± 0.045	0.023 ± 0.026	−0.006 ± 0.474	0.404	0.16	−0.022, 0.009
Knee flexion moment at TO, BW·BH	0.008 ± 0.005	0.008 ± 0.006	0.001 ± 0.004	0.240	0.19	−0.002, 0.001
Peak knee flexion moment, BW·BH	0.138 ± 0.026	0.104 ± 0.029	0.034 ± 0.031	<0.001 *	1.11	−0.044, −0.024
Peak knee extension moment, BW·BH	0.013 ± 0.006	0.011 ± 0.005	0.003 ± 0.006	0.011 *	0.43	0.001, 0.004
PVGRF, BW	1.14 ± 0.13	1.08 ± 0.13	0.06 ± 0.09	<0.001 *	0.64	−0.083, −0.027

* *p* < 0.05, reflecting interlimb differences. ILD, inter-leg difference. A positive value indicates the uninjured leg > injured leg; a negative value indicates the injured leg > uninjured leg; IC, initial contact; PVGRF, peak vertical ground reaction force; TO, toe-off; BW, body weight; BW·BH, body weight multiplied by body height.

**Table 5:** Correlation Analysis between Isokinetic Quadriceps Symmetry and Lower Limb Biomechanical Asymmetry.

Variables	Statistic	60°/s Qc	180°/s Qc	300°/s Qc	60°/s Qe
Hip flexion angle at IC, °	r (95% CI)	0.20 (−0.13, 0.48)	0.05 (−0.27, 0.36)	0.29 (−0.03, 0.55)	0.15 (−0.17, 0.45)
	*p* value	0.229	0.768	0.078	0.350
Knee flexion angle at PVGRF, °	r (95% CI)	−0.33 (−0.58, −0.02)	−0.21 (−0.50, 0.11)	−0.24 (−0.51, 0.09)	−0.43 (−0.66, −0.14)
	*p* value	0.041 *	0.192	0.150	0.006 *
Peak knee flexion angle, °	r (95% CI)	−0.39 (−0.63, −0.09)	−0.28 (−0.55, 0.04)	−0.29 (−0.56, 0.03)	−0.39 (−0.63, −0.09)
	*p* value	0.013 *	0.087	0.072	0.014 *
Knee flexion moment at PVGRF, BW·BH	r (95% CI)	−0.19 (−0.48, 0.13)	−0.38 (−0.62, −0.08)	−0.21 (−0.49, 0.12)	−0.13 (−0.43, 0.19)
	*p* value	0.240	0.017 *	0.211	0.418
Peak knee flexion moment, BW·BH	r (95% CI)	−0.20 (−0.48, 0.13)	−0.13 (−0.43, 0.19)	−0.25 (0.53, 0.07)	−0.01 (−0.32, 0.31)
	*p* value	0.234	0.424	0.119	0.966
Peak knee extension moment, BW·BH	r (95% CI)	−0.00 (−0.32, 0.32)	−0.01 (−0.32, 0.31)	−0.13 (−0.43, 0.19)	−0.01 (−0.327, 0.30)
	*p* value	0.995	0.966	0.433	0.936
PVGRF, BW	r (95% CI)	−0.09 (−0.39, 0.23)	−0.05 (−0.36, 0.27)	−0.12 (−0.42, 0.21)	−0.19 (−0.47, 0.14)
	*p* value	0.586	0.750	0.474	0.255

* *p* < 0.05. Qc, concentric quadriceps strength; Qe, eccentric quadriceps strength; PVGRF, peak vertical ground reaction force; BW, body weight; BW·BH, body weight multiplied by body height.

## Data Availability

The data supporting the findings of this study are not publicly available due to patient confidentiality and privacy law restrictions. However, they are available from the corresponding author upon reasonable request.

## Abbreviations

The following abbreviations are used in this manuscript:

ACLR	anterior cruciate ligament reconstruction.
ACL	Anterior Cruciate Ligament
QI	The quadriceps strength index
ILD	The inter-leg difference
IC	initial contact
TO	toe-off
PVGRF	Peak vertical ground reaction force
BW	body weight
BH	body height
BW·BH	body weight multiplied by body height
IKDC	International Knee Documentation Committee Subjective Knee Form
